# Ancestral function of the phytochelatin synthase C-terminal domain in inhibition of heavy metal-mediated enzyme overactivation

**DOI:** 10.1093/jxb/eraa386

**Published:** 2020-09-16

**Authors:** Mingai Li, Enrico Barbaro, Erika Bellini, Alessandro Saba, Luigi Sanità di Toppi, Claudio Varotto

**Affiliations:** 1 Department of Biodiversity and Molecular Ecology, Research and Innovation Centre, Fondazione Edmund Mach, San Michele all’Adige, Trento, Italy; 2 Dipartimento di Biologia, Università di Pisa, Pisa, Italy; 3 Dipartimento di Patologia Chirurgica, Medica, Molecolare e dell’Area Critica, Università di Pisa, Pisa, Italy; 4 Hasselt University, Belgium

**Keywords:** Cadmium, C-terminal domain, *Marchantia polymorpha*, overactivation, phytochelatin, phytochelatin synthase, site-directed mutagenesis, twin-cysteine motif, zinc

## Abstract

Phytochelatin synthases (PCSs) play essential roles in detoxification of a broad range of heavy metals in plants and other organisms. Until now, however, no *PCS* gene from liverworts, the earliest branch of land plants and possibly the first one to acquire a PCS with a C-terminal domain, has been characterized. In this study, we isolated and functionally characterized the first *PCS* gene from a liverwort, *Marchantia polymorpha* (*MpPCS*). *MpPCS* is constitutively expressed in all organs examined, with stronger expression in thallus midrib. The gene expression is repressed by Cd^2+^ and Zn^2+^. The ability of *MpPCS* to increase heavy metal resistance in yeast and to complement *cad1-3* (the null mutant of the Arabidopsis ortholog *AtPCS1*) proves its function as the only PCS from *M. polymorpha*. Site-directed mutagenesis of the most conserved cysteines of the C-terminus of the enzyme further uncovered that two twin-cysteine motifs repress, to different extents, enzyme activation by heavy metal exposure. These results highlight an ancestral function of the PCS elusive C-terminus as a regulatory domain inhibiting enzyme overactivation by essential and non-essential heavy metals. The latter finding may be relevant for obtaining crops with decreased root to shoot mobility of cadmium, thus preventing its accumulation in the food chain.

## Teaser

The characterization of *Marchantia polymorpha* phytochelatin synthase provides novel insights into the evolutionary function of the enzyme’s elusive C-terminus as a regulatory domain inhibiting enzyme overactivation by metal ions.

## Introduction

Plants are sessile organisms, thus they have evolved diverse defense mechanisms such as accumulation and detoxification of different metals to adapt to environmental stresses related to the mineral composition of soil. Some heavy metals such as zinc (Zn), copper (Cu), and iron (Fe) are essential for plant growth and development, as they are cofactors in protein structural and catalytic components, mediating ligand interactions and redox reactions (Giles *et al.*, 2003; Olson *et al.*, 2013; Schmidt and Husted, 2019). Other heavy metal(loid)s such as cadmium (Cd), arsenic (As), and lead (Pb) are non-essential, as they have no biological function in plants. On the contrary, they are toxic even at micromolar concentrations, because of their competition with endogenous metal cofactors for binding sites (Rea, 2012). Excess heavy metals can even lead to acute toxicity and plant death. Therefore, tight regulation of heavy metal accumulation in plants is an important mechanism to maintain plant fitness (Tennstedt *et al.*, 2009). In plants, phytochelatins (PC_n_) are among the most important and studied chelators for heavy metal detoxification (Clemens, 2019).

PC_n_ are non-ribosomally synthesized cysteine-containing peptides which have the general structure (γ-Glu-Cys)*n*-X (where *n*=2–5, and X is generally glycine) (Grill *et al.*, 1985). PC biosynthesis starts from glutathione (GSH) in a transpeptidase reaction catalyzed by phytochelatin synthase (PCS; EC 2.3.2.15) (Grill *et al.*, 1989). PC_n_ were discovered first in the fission yeast *Schizosaccharomyces pomb*e (Kondo *et al.*, 1984) and then in plants from cell cultures of *Rauvolfia serpentina* (Grill *et al.*, 1985). Afterwards, PC_n_ were identified in all plant species investigated as well as in algae, fungi, diatoms, and animals (Rea *et al.*, 2004; Tsuji *et al.*, 2004). *PCS* genes are constitutively expressed, and PC accumulation is activated by exposure to various physiological and non-physiological metal ions (Vatamaniuk *et al.*, 2000) and sequestrated into the vacuole through ATP-dependent transporters (Song *et al.*, 2010). Vacuole sequestration terminates the complexation of PC_n_ with heavy metals and prevents accumulation of heavy metal ions in the cytosol.

Cloning and functional characterization of *PCS* genes from *Arabidopsis thaliana* (*AtPCS1*), *S. pombe* (*SpPCS*), *Triticum aestivum* (*TaPCS1*), and *Caenorhabditis elegans* (*CePCS1*) (Glaeser *et al.*, 1991; Howden *et al.*, 1995; Clemens et al., 1999, 2001; Ha *et al.*, 1999; Vatamaniuk et al., 1999, 2001) dramatically increased our knowledge of how PC_n_ control heavy metal detoxification at the molecular level. In addition, the identification of PC-deficient mutants from Arabidopsis (*cad1*) and *S. pombe* further broadened our understanding of the roles of PC_n_ in heavy metal accumulation and tolerance. Based on these pioneer studies, later research attempted to increase heavy metal accumulation and tolerance in plants. A series of *PCS* genes from different species were isolated and overexpressed in model species (Liu *et al.*, 2011; Zhang *et al.*, 2018; Li *et al.*, 2019), but, surprisingly, these transgenic approaches resulted in diverse outcomes. For instance, transgenic plants, which were highly sensitive to Cd treatment, were obtained by overexpressing *AtPCS1* in Arabidopsis (Lee *et al.*, 2003a), even though there was only a small increase of PC_n_ compared with wild-type (WT) plants. On the other hand, overexpression of the same gene in *Brassica juncea* resulted in high tolerance to Cd and Zn exposure, and the accumulations of Cd and Zn were significantly lower than in WT plants (Gasic and Korban, 2007). Overall, it was estimated that 33.3% of experiments with transgenic plants overexpressing *PCS1* showed a positive relationship between Cd tolerance and accumulation, while 25% evidenced a negative relationship (Lee and Hwang, 2015). At present, the reasons underlying such contrasting effects are still not clear. The elegant works performed independently by different groups, however, indicate that the different response to Cd exposure of transgenic plants might be caused by several concurring factors, including differences of PCS activities, endogenous PC and GSH concentrations, as well as PC polymerization levels in transgenic plants (Wojas *et al.*, 2008; Brunetti *et al.*, 2011; De Benedictis *et al.*, 2018). Another important aspect intensively addressed was the elucidation of the structural bases of PCS function. Analysis of AtPCS1 by limited proteolysis showed that the conserved N-terminal domain is necessary and sufficient for enzyme catalytic activity, while the evolutionarily divergent C-terminal domain is involved in responsiveness to a wide range of heavy metals (Ruotolo *et al.*, 2004). The crystal structure of a cyanobacterial PCS homolog (lacking, like all cyanobacterial PCS enzymes, the C-terminal domain) suggested that the N-terminus is essential for core catalysis, and further implied the involvement of the C-terminal domain present in eukaryotes in sensing free heavy metals (Vivares *et al.*, 2005; Rea, 2006). The different responsiveness to a set of heavy metals of LjPCS1 and LjPCS3, two different PCS enzymes in *Lotus japonicus*, indicated that the different patterns of heavy metal activation between these two proteins were mainly due to the differences in their C-terminal domains (Ramos *et al.*, 2008). More recently, in Arabidopsis, the region of the AtPCS1 C-terminal domain responsible for Zn-dependent PC formation was identified through a set of C-terminal truncations (Kühnlenz *et al.*, 2016), while As-specific activation of PC synthesis was demonstrated to occur in another small region of the C-terminal domain (Uraguchi *et al.*, 2018).

Although the molecular mechanisms of heavy metal detoxification by PC_n_ from higher plant species have been studied intensively during the last decades, very few studies on early diverging plant lineages have been carried out. The constitutive presence of PCSs in some species of bryophytes and lycophytes was demonstrated through HPLC and MS analyses (Petraglia *et al.*, 2014; Bellini *et al.*, 2020b), and PC-mediated heavy metal detoxification was confirmed to be compartmentalized in vacuoles in the liverwort *Lunularia cruciata* (L.) Dumort (Degola *et al.*, 2014) and *Leptodictyum riparium* (Bellini *et al.*, 2020a). However, no isolation and detailed functional characterization of *PCS* genes from bryophytes have been reported to date. Bryophytes, comprising liverworts, mosses, and hornworts, are the earliest diverging lineages of land plants (Qiu *et al.*, 2006; Shimamura, 2016); so far, the phylogenetic relationship among these three bryophytes is still enigmatic (Shaw and Renzaglia, 2004), but liverworts are considered to be placed in a key phylogenetic position among the earliest land plants. The model species *Marchantia polymorpha* is a dioecious liverwort with separate female and male gametophytes that produce archegoniophores and antheridiophores, respectively. Due to its dominant haploid life cycle and easy asexual propagation through gemmae yielding isogenic experimental lines, *M. polymorpha* has well established molecular genetic tools ranging from mutant populations, genetic transformation, silencing, and genome editing (Ishizaki *et al.*, 2016). Furthermore, its genome was fully sequenced recently (Bowman *et al.*, 2017). Therefore, *M. polymorpha* has been widely used as a model species to elucidate the evolutionary processes of gene regulation mechanisms across land plants (Busch *et al.*, 2019; Naramoto *et al.*, 2019), but, to date, no systematic investigation has been performed on heavy metal detoxification in this species.

In this study, the putative *PCS* gene from *M. polymorpha* (*MpPCS*) was isolated and functionally characterized *in vivo* through overexpression in yeast and Arabidopsis to address the question of whether it is functional and can complement higher plants PCS enzymes. With MpPCS being the most basal PCS with a C-terminal domain in land plants, we further asked whether analysis of the few highly conserved cysteines in this region affect metal responsiveness to elucidate the ancestral function of this enigmatic domain.

## Materials and methods

### Plant materials, growth conditions, and stress treatments

*Marchantia polymorpha* L. Cam2 (UK Cambridge-2 WT) female gametophytes, *A. thaliana* Col-0 WT, and *Cad1-3* mutant and transgenic plants were used in this study. *Marchantia polymorpha* was propagated in Petri dishes containing half-strength Murashige and Skoog (MS) medium supplemented with 1% sucrose and 1% phytoagar under long-day conditions (16 h light/8 h dark) at 21 °C with a light intensity of 60 µmol m^–2^ s^–1^ in the growth chamber. For heavy metal treatments in *M. polymorpha*, 2-week-old gemmae were transferred to fresh Petri dishes either without or with addition of 50 µM CdSO_4_ or 200 µM ZnSO_4_. The gemmae were independently collected before and after treatments for 1, 3, 6, 12, and 24 h, snap-frozen in liquid nitrogen, and stored at –80 °C until used for real-time and thiol-peptide analyses. Three biological replicates at each sampling time point were applied for the entire treatment. For heavy metal treatments in Arabidopsis plants, sterilized seeds were germinated in half-strength MS agar medium and 2% (w/v) sucrose, supplemented either with 50 µM or 85 µM CdSO_4_, or with 200, 400, or 600 µM ZnSO_4_ in 100×100×15 mm square plates. In total, 15 seeds for Col-0 and each transgenic line were sown in one plate. After stratification at 4 °C for 3 d, the plates were grown vertically for 10 d under standard long-day conditions at 23 °C with a light intensity of 100–120 µmol m^–2^ s^–1^ in the growth chamber. At least 80 plants per genotype were processed for this analysis. For thiol-peptide analyses of *cad1-3* complementation lines, plants grown for 10 d on the control plates were transferred either to plates containing 85 µM CdSO_4_ or to fresh plates, and maintained for an additional 3 d. A total of 15–20 seedlings per genotype were pooled as a single biological sample, frozen in liquid nitrogen, and stored at –80 °C.

### Cloning, plasmid constructs, and transformation

For the analysis of the *MpPCS* expression pattern in *M. polymorpha*, a promoter region of 2.8 kb upstream of the *MpPCS* coding sequence (CDS) was amplified using primers MpPCS-prom_For and MpPCS-prom_Rev (see Supplementary Table S1 at *JXB* online) with Phusion High Fidelity DNA Polymerase (Thermo Scientific), cloned into pENTR/D TOPO vector (Invitrogen), and recombined into the destination vector pMpGWB104 (Ishizaki *et al.*, 2015) in front of the β-glucuronidase (GUS) CDS using LR clonase II (Invitrogen). The T-DNA was integrated into the *M. polymorpha* genome by *Agrobacterium tumefaciens*-mediated transformation as previously described (Kubota *et al.*, 2013). T_1_ transgenic plants were selected on half-strength solid Gambourg B5 medium supplemented with 10 mg l^–1^ hygromycin. The isogenic G_1_ lines from the T_1_ lines were obtained by subcultivating single gemmae, and gemmae generated from G_1_ lines (G_2_ generation) were used for experimental analyses.

For overexpression of *MpPCS* in *M. polymorpha*, the full-length *MpPCS* cDNA was amplified with primers *MpPCS_For* and *MpPCS_Rev* (Supplementary Table S1). The resulting PCR fragment was cloned into the pENTR/D TOPO vector (Invitrogen) and recombined into the destination vector pK7WG2 under the transcriptional control of the strong constitutive *Cauliflower mosaic virus* (CaMV) 35S promoter (Karimi *et al.*, 2002) in the same way as mentioned above to yield the final construct (p35S::MpPCS). This construct was transformed into *A. tumefaciens* strain GV3101-pMP90RK by electroporation and further transformed into the *A. thaliana* Col-0 ecotype by the floral dip method (Clough and Bent, 1998). T_1_ transgenic lines were screened on solid MS medium supplemented with 50 mg l^–1^ kanamycin. Homozygous single-copy T_3_ seeds from two selected lines were used for all downstream analyses.

Mutational analysis of MpPCS was carried out as follows. In total, six mutations targeting three positions with higly conserved cysteines were introduced into the *MpPCS* WT CDS using the Quikchange Site Directed Mutagenesis Kit (Stratagene) in the *M. polymorpha* pENTR_MpPCS plasmid. All primers used for mutagenesis are listed in Supplementary Table S1. The resulting entry vectors, respectively named pENTR_MpPCS_M1, pENTR_MpPCS_M2, pENTR_MpPCS_M3, pENTR_MpPCS_M4, pENTR_MpPCS_M5, and pENTR_MpPCS_M6, and the cognate WT pENTR_MpPCS plasmid were further recombined into the pYES-DEST52 vector (Invitrogen™) and transformed into Cd-sensitive *Saccharomyces cerevisiae* strain YK44 (*ura3-52 his3-200,* ∆*ZRCDCot1*, mating type α) using the lithium acetate method (Gietz and Schiestl, 2007). All sequences used for any of the constructs described above were verified by sequencing with a 96-capillary 3730xl DNA Analyzer (Thermo Scientific).

### Histochemical analysis of GUS expression in transgenic *Marchantia polymorpha*

About 10–15 gemmae of 17-day-old WT and transgenic *M. polymorpha* lines were incubated at 37 °C overnight in GUS assay solution as previously described (Gazzani *et al.*, 2009); the chlorophyll was cleared with a series of incubations in fresh 70% ethanol (v/v).

### Total RNA extraction, cDNA synthesis, and real-time PCR (RT-PCR) analyses

Total RNA was extracted from 100 mg of frozen plant material using a Spectrum Plant Total RNA Kit (Sigma-Aldrich®) following the manufacturer’s instructions, and treated with Amplification-Grade DNase I (Sigma-Aldrich®) for elimination of genomic DNA contamination. The integrity and quality analyses of extracted total RNA were performed in Bioanalyzer 2100 (Agilent Technologies), and cDNA was then synthesized with 1 μg of total RNA using SuperScript™III Reverse Transcriptase (Invitrogen™). Semi-quantitative RT-PCR (qRT-PCR) was carried out for different organs of *M. polymorpha* using the *MpACT* gene as a reference (Saint-Marcoux *et al.*, 2015) and using *ActinII* for Arabidopsis transgenic plants. For real-time analysis, qRT-PCR was conducted with Platinum® SYBR® Green qPCR SuperMix-UDG (Invitrogen) using *MpAPT* and *MpACT* as reference genes for *M. polymorpha* (Saint-Marcoux *et al.*, 2015) and *AtActII* and *AtEF1α* for Arabidopsis transgenic plants in a Bio-Rad C1000 Thermal Cycler detection system. Stability of reference genes was calculated with the RefFinder software (Xie *et al.*, 2012). All reactions for qRT-PCR analyses were performed in triplicate, and the 2^−ΔΔCT^ method was applied to calculate fold changes. Primer sequences are listed in Supplementary Table S1.

### Phylogenetic reconstruction

*Arabidopsis thaliana* PCS1 protein was blasted against the *M. polymorpha* MarpolBase ‘primary’ and ‘alternative’ (version 3.1, November 2015) protein databases (https://marchantia.info/tools/blast/plant/) using an E-value cut-off of 10^–5^. The *A. thaliana* PCS1 protein was further blasted against all angiosperm Phytozome 12 (Goodstein *et al.*, 2012) proteomes. The resulting hits were downloaded and representative sequences were selected based on protein completeness and phylogenetic distance from each other, to provide a representative sample of PCSs in plants. Proteins were aligned using the MAFFT online server (Katoh *et al.*, 2019), and regions with low homology were removed using the GBLOCKS server (Talavera and Castresana, 2007) with standard settings. The best-fitting model of protein evolution was selected with the online version of SMS (Lefort *et al.*, 2017) and this model was directly applied for maximum likelihood phylogenetic reconstruction using the PhyML online server (Guindon *et al.*, 2010) and aBayes approximate support branch estimates. The resulting tree was visualized with FigTree v1.4.4.

### Yeast complementation assay and induction for thiol-peptide analyses

A single colony from each transformant carrying either the WT construct (pYES52-MpPCS), one of the six mutations (pYES52-MpPCS_M1, pYES52-MpPCS_M2, pYES52-MpPCS_M3, pYES52-MpPCS_M4, pYES52-MpPCS_M5, pYES52-MpPCS_M6), or the pYES52 empty vector was cultured in YSD-U liquid medium overnight at 30 °C. Culture aliquots normalized to OD_600_=0.5 were pelleted and resuspended in 500 µl of YPGAL [1% (w/v) yeast extract, 2% (w/v) peptone, 2% (w/v) galactose] liquid medium and further diluted to 10^–1^, 10^–2^, and 10^–3^, and 5 µl from each aliquot were spotted on YPGAL solid medium supplemented with or without CdSO_4_/ZnSO4. Yeast growth was stopped after a 3 d incubation at 30 °C. For the *in vivo* assay of thiol-peptides, yeast cells at an OD_600_ of 0.1 were shaken overnight at 30 °C in YSD-U liquid medium, and protein expression was induced at an OD_600_ of 0.5 for 4 h by supplementing 2% galactose and 100 µM CdSO_4_. Afterwards, cells were harvested by centrifugation, washed twice with distilled water, snap-frozen in liquid nitrogen, and stored at –80 °C. All experiments were independently repeated four times.

### Generation of recombinant protein for MpPCS, its C-terminal mutations, and PCS activity assay

The CDSs of full-length MpPCS and the six mutants mentioned above were amplified from the corresponding pENTR clone used for plant transformation with primers listed in Supplementary Table S1 and cloned into the expression vector pET28a in-frame with an N-terminal 6×His-tag. The expression plasmids were transformed into *Escherichia coli* Rosetta™(DE3)pLysS cells, which were induced by adding 0.5 mM isopropyl-β-d-thiogalactopyranoside with overnight culture at room temperature. The cells were harvested by centrifugation and the soluble fraction of recombinant protein was purified as previously described (Fischer *et al.*, 2014), further desalted using a PD-10 desalting column (GE Healthcare), quantified with the Quant-iT Protein Assay Kit (Thermo Fisher Scientific), and assessed by 10% SDS–PAGE. The PCS activity assay was carried out as previously described (Ogawa *et al.*, 2010; Uraguchi *et al.*, 2017). In brief, the reaction mixture (100 μl) containing 200 mM HEPES–NaOH (pH 8.0), 10 mM 2-mercaptoethanol, 12.5 mM GSH, 100 μM Cd or 200 μM Zn, and 50 ng of recombinant PCS was incubated at 35 °C for 60 min, then terminated by the addition of 25 μl of 10% trifluoroacetic acid (TFA). The terminated reactions were maintained at 10 °C in the autosampler tray and immediately analyzed by HPLC-ESI-MS-MS to identify and quantify PC_n_ produced using the analytical method described in (Bellini *et al.*, 2019). For an accurate quantification, terminated reactions were diluted by a factor of 100 only for PC_2_.

### Analyses of thiol-peptides

*Marchantia polymorpha* and *A. thaliana* samples, previously stored at –80 °C, were extracted according to Bellini *et al.* (2019), whereas yeast cells were extracted following the protocol described in Ramos *et al.* (2008) with some modifications. Briefly, yeast cells were resuspended in 300 μl of the extraction buffer containing 0.1% (v/v) TFA, 0.5 mM DTPA (diethylenetriaminepentaacetic acid), and 200 ng ml^–1^ (glycine-^13^C_2_,^15^N)-labeled GSH and PC_2_ internal standards. All the other analyses and quantification of thiol-peptides were performed following the procedures detailed in Bellini *et al.* (2019). System control, data acquisition, and processing were carried out using AB Sciex Analyst® version 1.6.3 software.

### *Cad1-3* complementation

*Cad1-3* mutant plants were used for transformation by the floral dip method (Clough and Bent, 1998) using *Agrobacterium tumefaciens* GV3101-pMP90RK harboring a plant gene expression construct (p35S::MpPCS). Transformed seeds were selected on MS agar medium containing 50 mg l^–1^ kanamycin, and T_3_ homozygous seeds were used for complementation analyses.

### Statistical analyses

Data with one independent factor were analyzed using one-way ANOVA. Data with two independent factors were analyzed using two-way ANOVA. Tukey’s multiple comparison and least significant difference (LSD) tests were used to identify significant differences. Differences were considered significant if *P*≤0.05 in the two-sided test. Compact letter display was used to summarize the differences among means. All analyses were run in R version 4.0.0 (04.24; R Core Team, 2020) using the scripts provided in Mangiafico (2015). All experiments were perfomed with at least *n*=3 biological replicates.

## Results

### Phylogenetic reconstruction of MpPCS

**Fig. 1.**
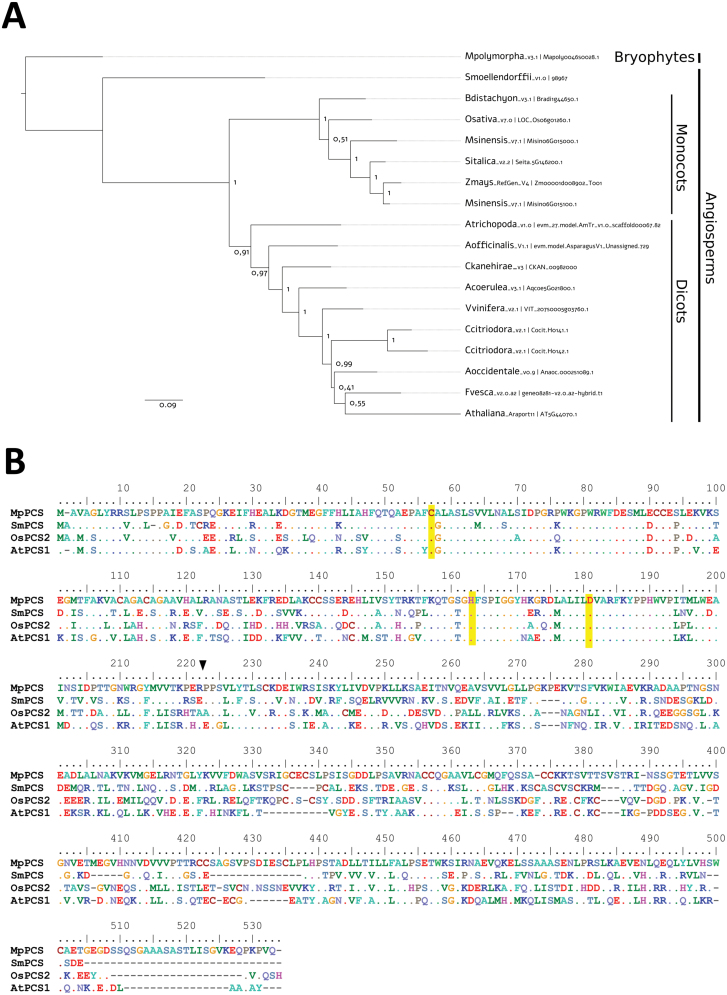
Phylogenetic reconstruction and multiple sequence alignment of PCS proteins. (A) Maximum likelihood tree of PCS proteins from representative plant taxa. Numbers are approximate Bayes (aBayes) support values calculated by PhyML. (B) Multiple sequence alignment of MpPCS (Mapoly0046s0028.1), SmoPCS (98967), OsaPCS (LOC_Os06g01260.1), and AtPCS (AT5G44070.1). Dashes (–) represent gaps; dots indicate residues identical to those of the first sequence. The amino acids of the catalytic triad (Cys56, His162, and Asp180 in *A. thaliana*, corresponding to the same positions in MpPCS) are highlighted with a yellow background. The boundary between the N-terminus (1–221 amino acids in *A.thaliana*) and the enzyme end is indicated with a black arrow. The color of the amino acids indicates the chemico-physical properties (e.g. red is used for negatively charged and blue for positively charged amino acids).

Based on a homology search of AtPCS1 in the fully sequenced genome of *M. polymorpha* (Bowman *et al.*, 2017), the full-length coding region of PCS of *M. polymorpha* (*MpPCS*) was identified and isolated (accession number Mapoly0046s0028.1). Only a single copy of *PCS* is present in the *M. polymorpha* genome, in contrast to the two copies found in Arabidopsis and many other higher plant species (Filiz *et al.*, 2019). The MpPCS protein is, as expected, basal to all angiosperm PCSs (Fig. 1A). *MpPCS* encodes a 530 amino acid polypeptide with a predicted molecular mass of ~57 kDa. The protein sequence alignment among MpPCS and other PCSs from higher plant species indicated that it shares 46–52% overall sequence identity (Fig. 1B). Conservation of the N-terminal domain is higher than that of the more divergent C-terminal domain, and the N-terminal domain has the typical catalytic triad of PCS enzymes, namely Cys56, His162, and Asp180 (Romanyuk *et al.*, 2006; Li *et al.*, 2019) (Fig. 1B).

### *MpPCS* is constitutively expressed and repressed by Cd^2+^ and Zn^2+^ treatment in vegetative organs of *M. polymorpha*

**Fig. 2.**
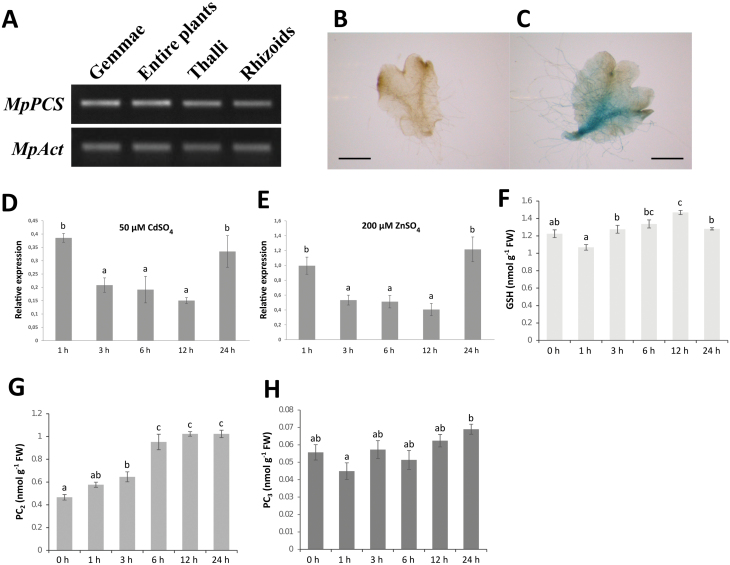
Expression pattern of *MpPCS* and thiol-peptide quantification. (A) Semi-quantitative RT-PCR of *MpPCS* transcription in different organs, using *MpAct* as an internal reference gene (33 PCR cycles for *MpPCS* and 27 for *MpAct*). Spatial expression pattern of 17-day-old wild-type (B) and transgenic *M. polymorpha* plants (C) by GUS staining. The ventral side of the thallus is shown; scale bars in the corner indicate 1 mm. Relative expression levels of *MpPCS* by qRT-PCR from 2-week-old *M. polymorpha* exposed to 50 µM CdSO_4_ (D) or 200 µM ZnSO_4_ (E) for different lengths of time as indicated. Bars indicates the SD (*n*=3 biological replicates) and different letters represent statistically significant differences (one-way ANOVA test, *P*<0.05). (F) GSH amount. (G) PC_2_ amount. (H) PC_3_ amount. Bars indicate the SE (*n*=4 biological replicates), and different letters represent statistically significant differences (two-way ANOVA test, *P*<0.05).

Semi-quantitative RT-PCR was performed to determine the expression patterns of *MpPCS* in *M. polymorpha* gemmae, entire plants, 4-week-old thalli, and rhizoids. This analysis indicated that *MpPCS* was expressed at similar levels in all four organs examined here (Fig. 2A). Furthermore, the expression pattern of *MpPCS* was visualized in transgenic *M. polymorpha* expressing the GUS gene under the control of the *MpPCS* promoter. The GUS staining analysis of 17-day-old gemmae showed that *MpPCS* was expressed mainly in the midrib region of the thallus and rhizoids; no expression was observed correspondingly in WT plants (Fig. 2B, C).

To assess the general responsiveness of *MpPCS* in *M. polymo*rpha to heavy metal treatments, 2-week-old gemmae were treated with either 50 µM CdSO_4_ or 200 µM ZnSO_4_, and the entire gemmae were collected at different time points before and after heavy metal treatments and subjected to qRT-PCR analysis. The overall stabilities of the two reference genes used, *MpACT* and *MpAPT*, were 1.41 and 1.19, respectively. The expression levels of the *MpPCS* transcript under CdSO_4_ and ZnSO_4_ treatments gradually decreased, reaching the minimum after 12 h of treatment, and then increased after 24 h to a level similar to that at 1 h of treatment (see Fig. 2D). One-way ANOVA further indicated that the expression level of *MpPCS* after 3, 6, and 12 h of treatment with both heavy metals was significantly different from those of the other time points tested (Fig. 2D, E). Given the identical trends for Zn^2+^ and Cd^2+^ treatments, we assessed the amount of GSH and PC_n_ only for Cd. The means of the GSH amounts over time were significantly different from those of control gemmae, as the amount of GSH had a slight increase at 12 h after CdSO_4_ treatment (one-way ANOVA, *F*_5,18_=12.992, *P*=1.898×10^–5^; Fig. 2F). On the other hand, PC_2_ content increased from 3 h on and reached a plateau at about double the amount of the control from 6 h to 24 h (one-way ANOVA, *F*_5,18_=40.267, *P*=3.713×10^–9^; Fig. 2G). The amount of PC_3_ did not significantly change as compared with the control, although significant differences could be found among time points (one-way ANOVA, *F*_5,18_=3.5281, *P*=0.0213; Fig. 2H).

### *MpPCS* overexpression confers heavy metal tolerance to YK44 yeast and hypersensitivity to Arabidopsis plants

Overexpression of candidate *PCS* genes in heterologous systems is a common method to assess their functionality and capacity to change responsiveness to heavy metals (Lee *et al.*, 2003b). First of all, *MpPCS* was transformed into yeast strain YK44, which is hypersensitive to heavy metal treatment. Growth of yeast lines transformed with either the *MpPCS* CDS or the empty vector was examined on YPGAL medium supplemented without and with different concentrations of Cd^2+^ and Zn^2+^. This analysis clearly showed that in the control medium (no heavy metals), growth of yeast transformed either with *MpPCS* or the empty vector was similar, while yeast expressing *MpPCS* grew more than the empty vector control line at concentrations of 100 µM CdSO_4_ and 700 µM ZnSO_4_ (Supplementary Fig. S1). Therefore, overexpression of *MpPCS* enhanced resistance of yeast strain YK44 to heavy metal stress.

**Fig. 3.**
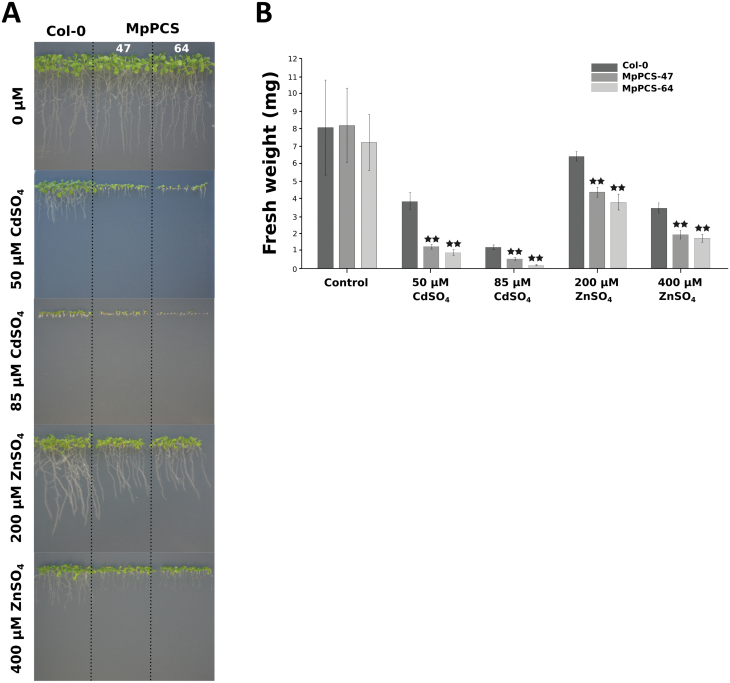
Phenotypic variations upon CdSO_4_ or ZnSO_4_ treatment of Arabidopsis transgenic plants overexpressing MpPCS from *M. polymorpha*. (A) Phenotypes of 10-day-old MpPCS transgenic and wild-type Col-0 plants under non-treated (top) and treated conditions with different concentrations of CdSO_4_ (50 µM and 85 µM; middle) or ZnSO_4_ (200 µM and 400 µM; bottom). Dashed lines indicate the separation of different genotypes. (B) Fresh weight of the corresponding plants shown in (A); bars represent the SD of three biological replicates, and two stars indicate statistically very significant differences compared with Col-0. At least 60 plants were used for each analysis.

In addition, transgenic Arabidopsis plants were generated to evaluate the ability of the 35S::MpPCS construct to affect tolerance to heavy metals *in planta*. Semi-quantitative RT-PCR was carried out first to estimate the relative expression of *MpPCS* in single-copy transgenic Arabidopsis lines (Supplementary Fig. S2), and the two lines with the highest relative expression were selected for phenotypic analyses. In control growth medium, both transgenic lines overexpressing *MpPCS* under the control of the strong *35S* promoter and Col-0 WT plants grew comparably, as no statistical differences were detected in fresh weight and root length among genotypes (Fig. 3A). However, when plants were grown in medium supplemented with 50 µM CdSO_4_, the mean fresh weights of both transgenic lines were significantly lower than that of Col-0 plants (Fig. 3B), and root length was also much shorter compared with Col-0. The same growth trend, in a more severe manner, was also observed for both transgenic lines and Col-0 plants treated with 85 µM CdSO_4_ (Fig. 3B). To assess whether the phenotypic variation observed for CdSO_4_ treatment would be the same also for an excess of an essential heavy metal, both transgenic lines and Col-0 WT plants were grown on the same basal medium with different concentrations of ZnSO_4_. Col-0 plants grew more than both transgenic lines under 200 µM ZnSO_4_ treatment (Fig. 3A), and both fresh weight and root length were significantly different between the two transgenic lines and Col-0 (Fig. 3B). Significant differences were also observed under 400 µM ZnSO_4_ treatment (Fig. 3B).

### Overexpressing *MpPCS* complements the Arabidopsis *Cad1-3* mutant

**Fig. 4.**
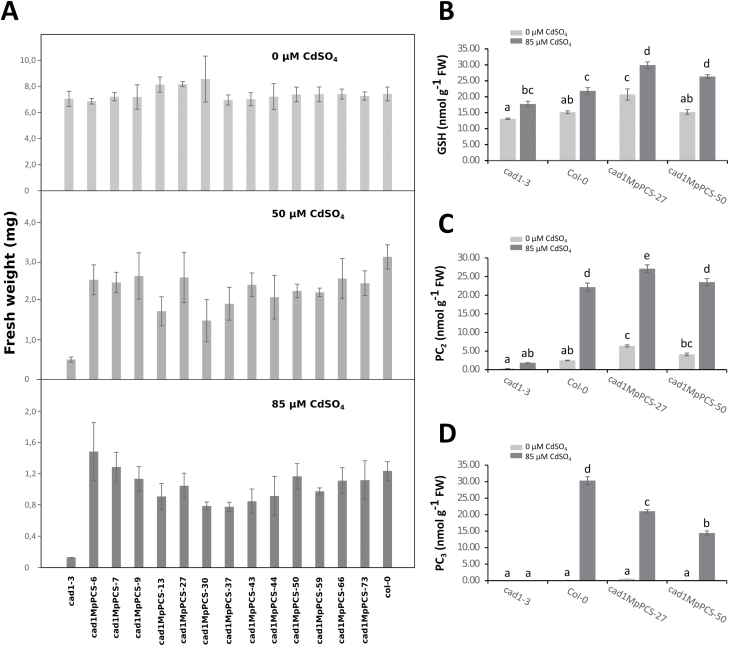
Functional complementation of the *cad1-3* mutant by overexpressing *MpPCS*. (A) Fresh weight was measured to evaluate the recovery of Cd^2+^ hypersensitivity from thirteen 10-day-old independent transgenic T_3_ lines compared with *cad1-3* and Col-0 plants growing in a medium supplemented with 0 µM (top), 50 µM (middle), or 85 µM (bottom) CdSO_4._ Bars correspond to the SD (*n*≥45 plants). (B) GSH amount. (C) PC_2_ amount. (D) PC_3_ amount. For thiol-peptide analyses, 10-day old seedlings were treated with or without 85 µM CdSO_4_ for an additional 3 d. Bars indicate the SE (*n*=4 biological replicates), and different letters represent statistically significant differences (two-way ANOVA test, *P*<0.05).

The *cad1-3* mutant, a knockout mutation of Arabidopsis *PCS1* (*AtPCS1*), is highly sensitive to treatment with heavy metals such as Cd^2+^ (Howden *et al.*, 1995; Ha *et al.*, 1999). Thus, to verify whether *MpPCS* was the functional *M. polymorpha* ortholog of *AtPCS1*, a complementation assay was performed by overexpressing *MpPCS* in the *cad1-3* mutant. In total, 13 independent homozygous lines were used to assess growth under different concentrations of CdSO_4_ treatment compared with Col-0 and the *cad1-3* mutant. In control growth medium, seedlings of all transgenic lines grew similarly compared with those of Col-0 and the *cad1-3* mutant, and no statistically significant differences in fresh weight were detected among all genotypes (Fig. 4). When the growth medium was supplemented with 50 µM or 85 µM CdSO_4_, the fresh weights of all lines and Col-0 were very similar from each treatment: statistical analysis indicated that no significant differences for the majority of lines were detectable, but many were significantly more resistant to CdSO_4_ treatment compared with the *Cad1-3* mutant. We then assessed the amount of GSH and PC_n_ for controls and plants treated with 85 µM CdSO_4_. A significant interaction was found among Cd^2+^ concentration and genotypes with respect to GSH concentration (two-way ANOVA, *F*_3,24_=4.5085, *P*=0.01206; Fig. 4B). The amount of GSH was significantly higher for the MpPCS-27 transgenic line compared with all other genotypes in control conditions, while both transgenic lines had a higher GSH amount under Cd^2+^ treatment. All genotypes had higher GSH amounts under Cd^2+^ treatment than under control conditions (Fig. 4B). A significant interaction was found among Cd^2+^ concentration and genotypes with respect to PC_2_ concentration (two-way ANOVA, *F*_3,24_=99.83, *P*=1.082×10^–13^; Fig. 4C). PC_2_ content did not change for the *cad1-3* genotype, but increased significantly for all other genotypes upon Cd^2+^ treatment. Both transgenic lines had levels of PC_2_ comparable with (MpPCS-50) or slightly higher (MpPCS-27) than Col-0 (Fig. 4C). A significant interaction was also found among Cd^2+^ concentration and genotypes with respect to PC_3_ concentration (two-way ANOVA, *F*_3,24_=308.66, *P*<2.2×10^–16^; Fig. 4D), with a similar trend to that of PC_2_, but in this case Col-0 produced more PC_3_ than both transgenic lines (Fig. 4D). To confirm whether phenotypic complementation was due to overexpression of the *MpPCS* gene, the expression level was measured by qPCR for all tested lines. Even though variations of expression levels were detected, *MpPCS* was expressed in all 13 lines (Supplementary Fig. S3).

### Diverse heavy metal responsiveness in C-terminal point mutations of MpPCS in yeast and PCS activity assay of recombinant protein

**Fig. 5.**
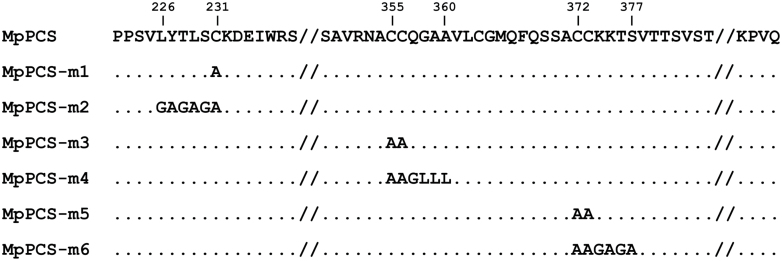
Scheme of C-terminal mutations in MpPCS proteins. The numbers above the wild-type sequence indicate the first and last positions of mutated amino acids, a dot indicates any amino acid identical to that of wild-type MpPCS, and the symbol ‘//’ marks the omission of a partial amino acid sequence due to space limitations.

**Fig. 6.**
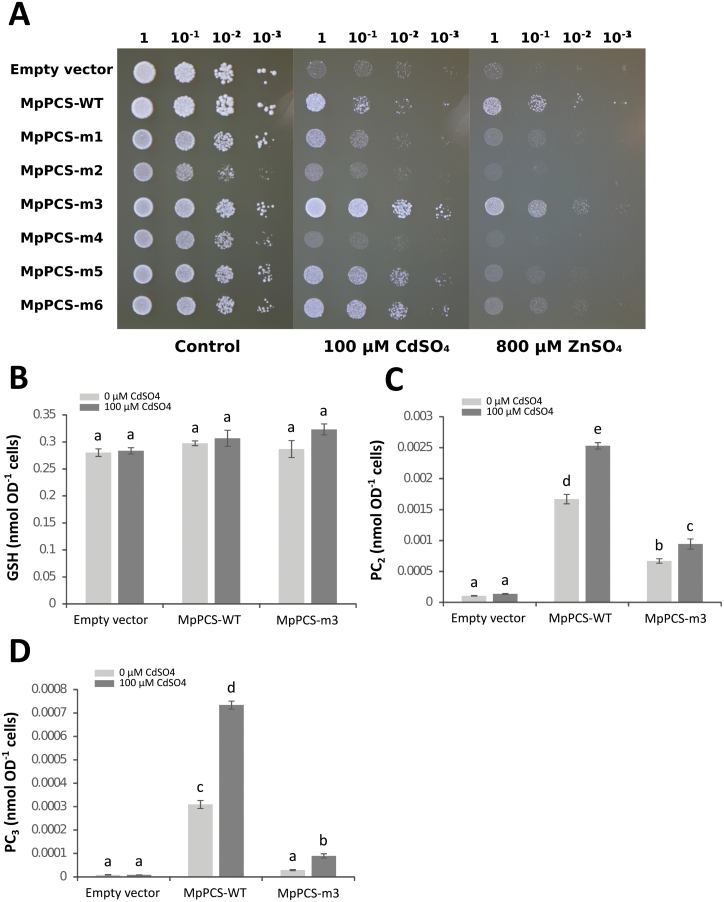
Yeast complementation assay and thiol-peptide quantification. (A) Yeast growth comparison of the heavy metal-hypersensitive strain YK44 transformed with empty vector, wild-type MpPCS (MpPCS-WT), and C-terminal mutagenized MpPCS (MpPCS-m1, MpPCS-m2, MpPCS-m3, MpPCS-m4, MpPCS-m5, and MpPCS-m6) growing in YPGAL medium supplemented with 0 µM, 100 µM CdSO_4_, or 800 µM ZnSO_4_. Dilution factors are shown above each picture. (B) GSH amount. (C) PC_2_ amount. (D) PC_3_ amount. For thiol-peptide analyses, yeast cells were treated with or without 100 µM CdSO_4_ for 4 h. Bars indicate the SE (*n*=4 biological replicates), and different letters represent statistically significant differences (two-way ANOVA test, *P*<0.05).

To pinpoint whether the amino acids in the C-terminal domain of MpPCS had a role in sensing of different heavy metals, six independent sets of mutations were constructed, targeting three different positions with evolutionarily conserved cysteines (mutants MpPCS-m1, MpPCS-m2, MpPCS-m3, MpPCS-m4, MpPCS-m5, and MpPCS-m6; Fig. 5). Upon transformation into yeast strain YK44, heavy metal responsiveness was first qualitatively evaluated on the growth medium following exposure to Cd^2+^ and Zn^2+^. In control YPGAL medium, yeast lines transformed with all six mutant constructs, WT MpPCS, and empty vector grew similarly, indicating that none of the constructs caused any effect on yeast in the absence of excess heavy metals (Fig. 6). Upon exposure to 100 µM Cd^2+^, the six constructs displayed variable levels of resistance to Cd^2+^. The yeast line transformed with MpPCS-m3 showed the highest resistance to Cd^2+^, being even more resistant than the line expressing WT MpPCS. On the other hand, the MpPCS-m4 line was less resistant than the MpPCS line, with growth levels similar to the empty vector line. The other mutations showed levels of resistance similar to the WT MpPCS line. Also in the case of Zn^2+^ treatment, the mutant lines showed different resistance patterns. Again, the MpPCS-m3 mutation caused higher tolerance to Zn^2+^ treatment than WT MpPCS. MpPCS-m1, MpPCS-m5, and Mpo-PCS-m6 lines were also resistant to Zn^2+^, but less so than the MpPCS line. The other mutations caused an almost complete loss of resistance to Zn^2+^, with growth rates very similar to that of the empty vector line. Given the identical trends for Zn and Cd treatments, we assessed the amount of GSH and PC_n_ only for Cd in the yeast lines transformed with the empty vector, the WT MpPCS, and the MpPCS-m3 mutant. In the case of GSH concentration, no significant interaction was found between the amount of Cd^2+^ and genotypes (two-way ANOVA, *F*_2,18_=1.380, *P*=0.277; Fig. 6B), and no significant difference was found in GSH content between Cd^2+^ concentrations and among genotypes (two-way ANOVA for Cd^2+^ treatments, *F*_2,18_=2.846, *P*=0.084). Thus, all the strains contained, under both control and treated conditions, the same amount of GSH (Fig. 6B). In the case of PC_2_, we found an interaction among amount of Cd^2+^ and genotypes (two-way ANOVA, *F*_2,18_=33.161, *P*=9.203×10^–7^; Fig. 6C). As expected, only trace amounts of PC_2_ were present in lines transformed with the empty vector either in the absence or in presence of 100 µM Cd^2+^. Significantly larger amounts of PC_2_ was present in lines transformed with either the WT MpPCS or the MpPCS-m3 constructs, with the latter containing less PC_2_ than the former in both control and treated conditions (Fig. 6C). In the case of PC_3_, the results were similar [interaction among amount of Cd^2+^ and genotypes, two-way ANOVA, *F*_2,18_=235.95, *P*=1.22×10^–13^ with the difference that the mean PC_3_ amount in MpPCS-m3 did not differ from those in lines transformed with the empty vector (Fig. 6D).

**Fig. 7.**
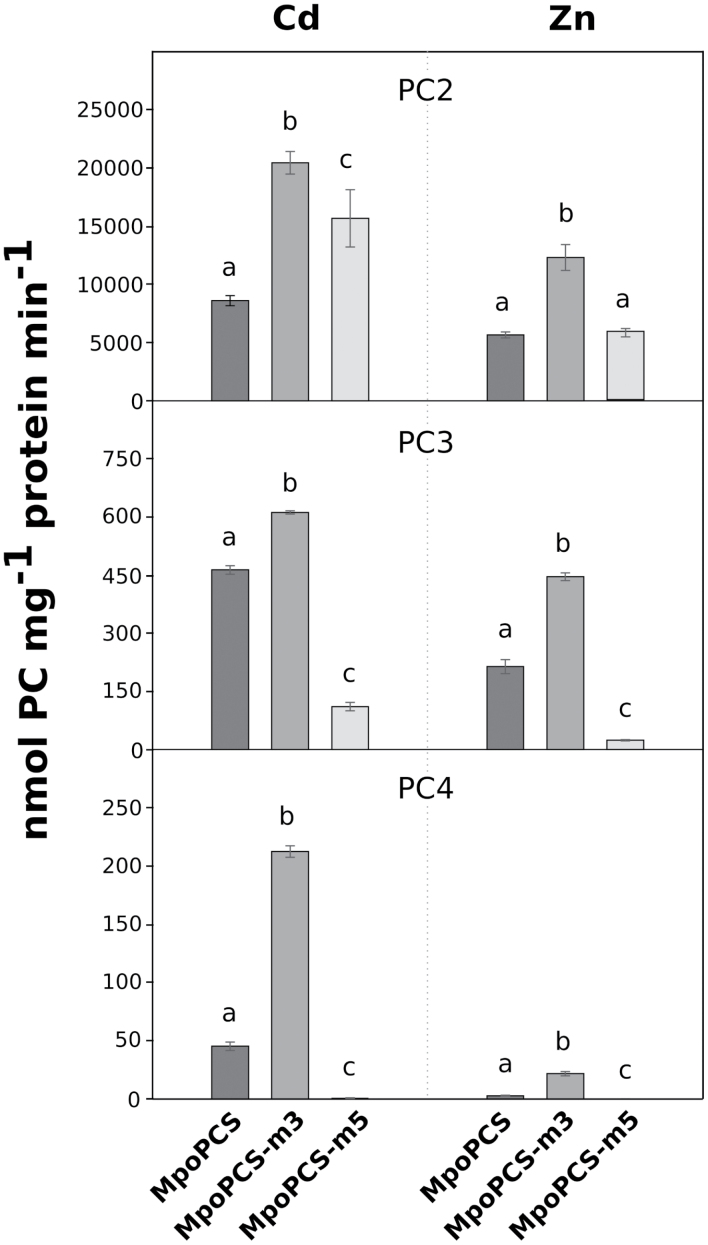
PC_n_ production by recombinant proteins MpPCS, MpPCS-m3, and MpPCS-m5 *in vitro*. Average PC_2_ (top), PC_3_ (middle), and PC_4_ (bottom) production was measured by activation with 100 µM CdSO_4_ or 200 µM ZnSO_4_ (indicated above the picture) in a reaction containing 500 ng ml^–1^ MpPCS, MpPCS-m3, or MpPCS-m5 proteins purified from *E. coli*. Bars correspond to the SD of the means. Five replicates were used for these analyses. The same letters above the bars represent no significant differences from each other (Tukey–Kramer test, *P*>0.05).

To confirm the qualitative differences observed in yeast in responses of the six mutations to Cd^2+^ and Zn^2+^ relative to wild-type MpPCS, we expressed all constructs as 6×His tag N-terminal fusions in *E. coli*. Soluble recombinant proteins could be purified only for three constructs (WT MpPCS, MpPCS-m3, and MpPCS-m5; Supplementary Fig. S4). For two other constructs (MpPCS-m2 and MpPCS-m4), proteins were expressed exclusively in inclusion bodies, while in the case of MpPCS-m6, no recombinant protein was expressed either in inclusion bodies or in the soluble supernatant in *E. coli*, suggesting that the mutations involving stretches of several amino acids could affect protein folding/stability. Thus, PCS activity could be measured exclusively for WT MpPCS, MpPCS-m3, and MpPCS-m5 enzymes upon Cd^2+^ and Zn^2+^ activation through quantification of the three major PC polymerization levels (PC_2_, PC_3_, and PC_4_). In the presence of 100 µM CdSO_4_, the means of total PC_n_ formation were significantly heterogeneous across the three enzymes (one-way ANOVA, *F*_2,12_=62.3, *P*=4.58×10^–7^), and it was significantly higher in both MpPCS-m3 and MpPCS-m5 enzymes compared with WT MpPCS in the order MpPCS-m3>MpPCS-m5>MpPCS (Supplementary Fig. S5). In the presence of 200 µM ZnSO_4_, the production of PC_n_ was still significantly heterogeneous across the three enzymes (one-way ANOVA, *F*_2,12_=131.6, *P*=6.86×10^–9^). Also in this case, the highest PC_n_ production among them was from MpPCS-m3, while MpPCS did not differ significantly from MpPCS-m5 (Supplementary Fig. S5). In addition, to evaluate in more detail the pattern of individual PC_n_ production, the comparison among different genotypes upon heavy metal induction was examined. For the 100 µM CdSO_4_ treatment, the activity of MpPCS-m3 was significantly higher than those of MpPCS and MpPCS-m5 for the production of PC_2_ and PC_3_, as well as PC_4_ (Fig. 7). The activity of MpPCS-m5 was also significantly higher than that of MpPCS for PC_2_, but significantly lower than MpPCS for PC_3_ and PC_4_ production. The same pattern was also observed for MpPCS-m3 subjected to ZnSO_4_ treatment, and also for MpPCS-m5 only for PC_3_ and PC_4_ formation; however, no significant difference between MpPCS and MpPCS-m5 was detected for PC_2_ (Fig. 7). Thus, the MpPCS-m3 and MpPCS-m5 mutations significantly varied in the polymerization level of PC_n_ as compared with WT MpPCS, with MpPCS-m3 having a higher polymerization level and MpPCS-m5 having a lower polymerization level than the WT in both heavy metal treatments.

## Discussion

Decades of studies have shown that PC_n_ are constitutively present in various plant lineages and play critical roles for detoxification/homeostasis of a wide range of heavy metals (Degola *et al.*, 2014; Kühnlenz *et al.*, 2016; Fontanini *et al.*, 2018). Till now, however, only the genes encoding PCS proteins in higher plant species have been isolated and functionally characterized (e.g. Loscos *et al.*, 2006; Li *et al.*, 2019). In contrast, no detailed molecular studies of PCSs of early diverging land plants such as liverworts have been conducted, despite the high relevance of this clade which is considered to encompass the earliest representatives of the radiation of plants on land (Bowman *et al.*, 2016). Thus, in this study, we addressed the functional characterization of MpPCS from *M. polymorpha* to shed new light on the evolution of molecular mechanisms for heavy metal detoxification in land plants and in particular on the role of the PCS C-terminal domain.

### *MpPCS* is the functional ortholog of angiosperm *PCS* genes

Several lines of evidence indicate that *MpPCS* shares common features with the *PCS* genes characterized in angiosperms so far. MpPCS has the two domains found in all other PCS proteins, a typically highly conserved N-terminal domain and a more divergent C-terminal domain. MpPCS further displays in its N-terminal domain fully conserved amino acids of the catalytic triad (Fig. 1B; Romanyuk *et al.*, 2006). The *MpPCS* gene is constitutively expressed in different organs under control growing conditions (Fig. 2A), and its overexpression in yeast strain YK44 enhances heavy metal resistance upon exposure to Cd^2+^ or Zn^2+^ like many other functional PCSs (Supplementary Fig. S1; Liu *et al.*, 2011; Zhao *et al.*, 2014). Furthermore, overexpression of the *MpPCS* CDS fully complements the PC-deficient mutant *cad1-3* in Arabidopsis (Fig. 4). Additionally, the purified recombinant protein MpPCS was able to catalyze the synthesis of PC_n_ using GSH as substrate following activation by Cd^2+^ or Zn^2+^*in vitro* (Fig. 7; Supplementary Fig. S5). Taken together, these data clearly demonstrate that *MpPCS* is the ortholog in *M. polymorpha* and has the same function as *AtPCS1*.

Previous studies have shown transcriptional regulation of angiosperm *PCS* genes upon heavy metal exposures. For instance, the transcripts of *AtPCS1* in Arabidopsis young seedlings were induced upon heavy metal Cd^2+^ exposure (Lee and Korban, 2002). Also, a dramatic increase of *TaPCS1* expression in wheat root was observed after Cd^2+^ treatment (Clemens *et al.*, 1999), and the time course analyses of *MaPCS1* and *MaPCS2* expression patterns in different organs of mulberry also indicated significant induction upon exposure to Cd^2+^ or Zn^2+^ (Fan *et al.*, 2018). Interestingly, in contrast to these results, the expression of *MpPCS* was repressed upon Cd^2+^ and Zn^2+^ exposure. Analogously to higher plant *PCS* genes, however, MpPCS induction by Cd^2+^ was modest, attaining just a 2-fold change as compared with untreated controls (Lee and Korban, 2002; Li *et al.*, 2019), confirming the overall limited transcriptional responsiveness of PCS in the course of evolution. This observation further suggests that the well-known heavy metal-dependent post-transcriptional regulation of PCS enzymatic activity has been playing a major role in the tight control of PC_n_ production since the early stages of evolution of this enzyme. Our results on both the differential transcriptional regulation and enzymatic activation by Cd^2+^ as compared with Zn^2+^ further confirm the highest induction capacity of the former to elicit PC_n_ biosynthesis, as previously suggested not only for higher plants but also for another liverwort, *Lunularia cruciata* (L.) Dumort (Degola *et al.*, 2014; Petraglia *et al.*, 2014).

Many studies have attempted to increase the heavy metal tolerance of plants by overexpressing *PCS* genes from different species, but only in a minority of cases have enhanced tolerance to heavy metals and increased PC_n_ content been attained (Liu *et al.*, 2012; Shukla *et al.*, 2012; Fan *et al.*, 2018). More commonly, overexpression of heterologous *PCS* genes resulted in hypersensitivity to heavy metals (Wojas *et al.*, 2008; Wang *et al.*, 2012; Li *et al.*, 2019). In the case of transgenic plants overexpressing *MpPCS* in Arabidopsis, a substantial increase of sensitivity to Cd^2+^ or Zn^2+^ compared with WT Col-0 was obtained (Fig. 3A, B). Almost certainly this result can be explained by a depletion of the pool of GSH, the substrate of PCS enzymes, and the resulting disruption of the cellular redox balance (Wojas *et al.*, 2008; Brunetti *et al.*, 2011). Taken together, our results highlight how, despite hundreds of million of years of divergent evolution between extant liverworts and angiosperms, the involvement of PCS in heavy metal detoxification is fully functionally conserved between *M. polymorpha* and *A. thaliana*.

### A conserved cysteine motif in the land plant PCS C-terminal domain prevents enzyme overactivation by heavy metals

The C-terminal domain of PCS has been demonstrated to be fundamental for the sustained PCS activity and stability necessary for plants to cope with elevated concentrations of the non-essential Cd^2+^ ion and proposed to be involved in heavy metal perception/specificity as well as metallochaperone and metallothionein functions (Ruotolo *et al.*, 2004; Vestergaard *et al.*, 2008). Identification of MpPCS as the functionally validated enzyme from the most basal land plant sequenced to date provides a unique opportunity to functionally test the ancestral role of this still enigmatic domain, as conserved cysteines have been proposed to be those most relevant for metal binding (Maier *et al.*, 2003). Among the three sets of most conserved cysteines in the C-terminus of land plants PCSs, two (C355–C356 and C372–C373) were homologous to residues previously identified to bind Cd^2+^ in TaPCS1 (C351–C352 and C369–C370), while C231 did not bind any metal ion (Maier *et al.*, 2003). While, in general, in our work the mutations involving multiple substitutions in a stretch of residues around the conserved cysteines (MpPCS-m2, MpPCS-m4, and MpPCS-m6) impaired protein solubility/stability too heavily to provide useful information, mutations of only the conserved cysteines (MpPCS-m1, MpPCS-m3, and MpPCS-m5) were highly informative. Consistent with a possible structural role, the C231A (MpPCS-m1) single mutation provided a very weak increase in heavy metal tolerance in yeast and could not be stably expressed in *E. coli*, suggesting that it is essential for correct folding/stability of the enzyme. In contrast, the other two double mutant proteins, C355A–C356A (MpPCS-m3) and C372A–C373A (MpPCS-m5), could be solubly expressed in *E. coli*, suggesting that they do not overly destabilize the enzyme. Indeed, the quadruple mutant of AtPCS1, where both cysteine homologs to C372A–C373A and an additional two cysteine residues in close proximity (not conserved in MpPCS) were mutated to alanines, was previously demonstrated to be fully stable (Vestergaard *et al.*, 2008). *In vivo* yeast expression does not support that the MpPCS-m3 mutation enhances PC_n_ production as compared with the WT MpPCS, indicating that in this heterologous system the MpPCS-m3 mutant enzyme is less active than the WT enzyme in the conditions tested. Currently the reasons for this difference remain to be investigated. However, *in vitro* enzymatic assays demonstrate that MpPCS-m5 and especially MpPCS-m3 mutations enhance PC_n_ production as compared with the WT enzyme. To the best of our knowledge, this is the first time a mutation increasing the enzyme Cd^2+^-responsive activity has been found. Recently, deletion of the last 10 amino acids of the AtPCS1 C-terminus was found to increase As^3+^-dependent PC_n_ production, suggesting that some of the residues in this region may inhibit PCS activation by As^3+^ (Uraguchi *et al.*, 2018). Our findings indicate that in addition to As^3+^-, Cd^2+^-dependent repressing mechanisms also possibly exist to prevent enzyme overactivation. Most importantly, these regulatory functions of the PCS C-terminus are evolutionarily conserved and can have different degrees of selectivity: while the MpPCS-m5 mutation selectively abolishes Cd^2+^-dependent repression, MpPCS-m3 mutation abolishes both Cd^2+^- and Zn^2+^-dependent repression *in vitro*. We speculate that the cysteines at both sites may constitute low-affinity binding sites for Cd^2+^ and Zn^2+^ that, above a certain threshold concentration of these metal ions, act by inhibiting PCS activity. A protective function of the C-terminus had been previously proposed in cases where the free heavy metal ion concentration would exceed those required for formation of the metal–thiolate substrate (Romanyuk *et al.*, 2006; Rea, 2012). AtPCS1 activity as a function of Cd^2+^ concentrations is indeed bell-shaped, with a maximum at ~1 µM (Rea, 2012), implying either heavy metal-mediated enzyme inactivation or the existence of feedback inhibition mechanisms to avoid enzyme overactivation in the presence of high heavy metal concentrations. Our results indicate that such mechanisms indeed exist, are conserved across the whole evolutionary history (hundreds of million of years of divergent evolution between extant liverworts and angiosperms; Rubinstein *et al.*, 2010) of land plants, and are mediated by metal-binding cysteines. This finding points to a likely role for the PCS C-terminal domain in maintaining homeostatic levels of essential ions (Steffens *et al.*, 1986; Grill *et al.*, 1987; Kühnlenz *et al.*, 2016) and GSH (Lee *et al.*, 2003a), while preventing Cd^2+^ toxicity. From an applied perspective, if confirmed *in planta*, the higher activity of PCS mutants with increased activity may be exploited to decrease the root to shoot transport of Cd^2+^, as previously suggested in the case of arsenic (Uraguchi *et al.*, 2018). This, in turn, would contribute to reduce the amount of this highly toxic heavy metal in the human food chain.

## Supplementary data

The following supplementary data are available at *JXB* online.

Table S1. List of primers used in this study.

Fig. S1. Yeast growth of heavy metal-hypersensitive strain YK44 transformed with empty vector and wild-type *MpPCS*.

Fig. S2. Semi-quantitative RT-PCR of *MpPCS* transcription in 16 independent Arabidopsis transgenic lines.

Fig. S3. Relative expression of *MpPCS* by qRT-PCR from 7-day-old seedlings of 13 transgenic Arabidopsis lines.

Fig. S4. Recombinant proteins MpPCS-WT, MpPCS-m3, and MpPCS5 purified from *E. coli* and electrophoresed by 10% SDS–PAGE.

Fig. S5. Total PC (PC_2_, PC_3_, and PC_4_) production by recombinant proteins MpPCS, MpPCS-m3. and MpPCS-m5 *in vitro*.

eraa386_suppl_Supplementary_Material
