# Supplementary material for: Ancestral function of the phytochelatin synthase C-terminal domain in inhibition of heavy metal-mediated enzyme overactivation
Source: J Exp Bot. 2020 Sep 16;71(20):6655–69. doi: 10.1093/jxb/eraa386 (PMC7586750; doi:10.1093/jxb/eraa386)
Supplement: eraa386_suppl_Supplementary_Material [file eraa386_suppl_supplementary_material.pdf]

Ancestral function of phytochelatin synthase C-terminal domain in inhibition of heavy metal-mediated enzyme overactivation

Mingai Li[1], Enrico Barbaro[1], Erika Bellini[2], Alessandro Saba[3], Luigi Sanità di Toppi[2#], Claudio Varotto[1#]

[1]Department of Biodiversity and Molecular Ecology, Research and Innovation Centre, Fondazione Edmund Mach, 38100, San Michele all'Adige (TN), Italy

[2]Dipartimento di Biologia, Università di Pisa, 56126, Pisa, Italy

[3]Dipartimento di Patologia Chirurgica, Medica, Molecolare e dell'Area Critica, Università di Pisa, 56126, Pisa, Italy

authors for correspondence.

Luigi Sanità di Toppi, Dipartimento di Biologia, Università di Pisa, via Luca Ghini 13, 56126, Pisa, Italy. E-mail:luigi.sanita@unipi.it

Claudio Varotto, Department of Biodiversity and Molecular Ecology, Research and Innovation Centre, Fondazione Edmund Mach. Via Mach 1, 38100, San Michele all'Adige (TN), Italy

Supplementary tables and figures

Table S1: list of primers used in this study.

Figure S1: Yeast growth of heavy metal hypersensitive strain YK44 transformed with empty vector and wild-type *MpPCS*. Series dilutions (indicated above the picture) of yeast cell with the same volume at OD_{600} = 0.5 were spotted on solid YPGAL medium supplemented with 0 µM, 50 µM and 100 µM $CdSO_4$ (top) or 400 µM, 700 µM and 1000 µM $ZnSO_4$ (bottom).

Figure S2: Semi-quantitative rt-PCR of *MpPCS* transcription in 16 independent Arabidopsis transgenic lines. *AtAct2* was used as an internal reference gene. 33 cycles were used for *MpPCS* amplification and 26 for *AtAct2*.

Figure S3: Relative expression of *MpPCS* by qRT-PCR from 7-day-old seedlings of 13 transgenic Arabidopsis lines. The Cd hypersensitive mutant *Cad1-3* was used to transform MpPCS_CDs driven

by the Cauliflower mosaic virus promoter (CaMV 35S). Bars indicate the standard deviation of the means.

Figure S4: Recombinant proteins MpPCS-WT, MpPCS-m3 and MpPCS5 purified from *E. coli* and electrophoresed in 10% SDS-PAGE. The first lane is the protein size marker, two different sizes (50 kDa and 75 kDa) are shown for the corresponding bands in the marker.

Figure S5: Total PC (PC2, PC3 and PC4) productions by recombinant proteins MpPCS, MpPCS-m3 and MpPCS-m5 *in vitro*. Average PCn productions were measured upon enzyme activation with 100 µM $CdSO_4$ (top) or 200 µM $ZnSO_4$ (bottom) in a reaction containing 500 ng/ml of MpPCS, MpPCS-m3 or MpPCS-m5 proteins purified from *E. coli*. Bars correspond to the standard deviation of the means. Five replicates were used for these analyses. Equal letters above the bars represent no significant differences from each other (Tukey-Kramer test, $p > 0.05$).

Table S1

Primer name	Sequence (5' - 3')	Purpose
MpPCS-prom_For	CACCGACGAGAATTGGGCCGCAAGA	cloning
MpPCS-prom_Rev	GCCTCCGTTGGTCGTTGTGTAAACTTG	cloning
MpPCS_For	CACCAACGGAGGCAAAATGGCGGT	cloning
MpPCS_Rev	CTACTGCACCGGCTTTGGCTGC	cloning
MpPCS-m1_For	CCGTCAGTTCTTTACACACTGTCTGCCAAGGATGAGATCTGGAGATCTAT	cloning
MpPCS-m1_Rev	ATAGATCTCCAGATCTCATCCTTGGCAGACAGTGTGTAAAGAACTGACGG	cloning
MpPCS-m2_For	CAAGCCGGAGCGACCTCCGTCAGTTGGTGCCGGAGCTGGAGCCAAGGATGAGATCTGGAGATCTATATC	cloning
MpPCS-m2_Rev	GATATAGATCTCCAGATCTCATCCTTGGCTCCAGCTCCGGCACCAACTGACGGAGGTCGCTCCGGCTTG	cloning
MpPCS-m3_For	CTTCCCTCAGCTGTCAGAAATGCTGCCGCTCAAGGGGCAGCTGTGTTGTGTGG	cloning
MpPCS-m3_Rev	CCACACAACACAGCTGCCCCTTGAGCGGCAGCATTTCTGACAGCTGAGGGAAG	cloning
MpPCS-m4_For	CTTCCCTCAGCTGTCAGAAATGCTGCCGCTGGATTGCTGCTTGTGTTGTGTGGGATGCAATTCCA	cloning
MpPCS-m4_Rev	TGGAATTGCATCCCACACAACACAAGCAGCAATCCAGCGGCAGCATTTCTGACAGCTGAGGGAAG	cloning
MpPCS-m5_For	GGGATGCAATTCCAGTCCTCTGCCGCTGCTAAGAAAACTTCAGTGACCACTTC	cloning
MpPCS-m5_Rev	GAAGTGGTCACTGAAGTTTTCTTAGCAGCGGCAGAGGACTGGAATTGCATCCC	cloning
MpPCS-m6_For	GGGATGCAATTCCAGTCCTCTGCCGCTGCTGGAGCTGGAGCCGTGACCACTTCGGTGAGCACTAGA	cloning
MpPCS-m6_Rev	TCTAGTGCTCACCGAAGTGGTCACGGCTCCAGCTCCAGCAGCGGCAGAGGACTGGAATTGCATCCC	cloning
MpPCS_RT_For	GATGTGAATGTAGTCTGCCGT	RT-PCR
MpPCS_RT_Rev	GTGAACTCCCTCCATAGTTTCC	RT-PCR
MpACT_RT_For	AGGCATCTGGTATCCACGAG	RT-PCR
MpACT_RT_Rev	ACATGGTCGTTCCTCCAGAC	RT-PCR
AtActII_RT_For	GCACCCTGTTCTTCTTACC	RT-PCR
AtActII_RT_Rev	AACCCTCGTAGATTGGCACA	RT-PCR
MpAPT_RT_For	CGAAAGCCCAAGAAGCTACC	RT-PCR
MpAPT_RT_Rev	GTACCCCCGGTTGCAATAAG	RT-PCR
MpPCS_pET28a_For	CGGATCCATGGCGGTTGCCGGGCTCTA	cloning
MpPCS_pET28a_Rev	AAGCTTCTACTGCACCGGCTTTGGCT	cloning

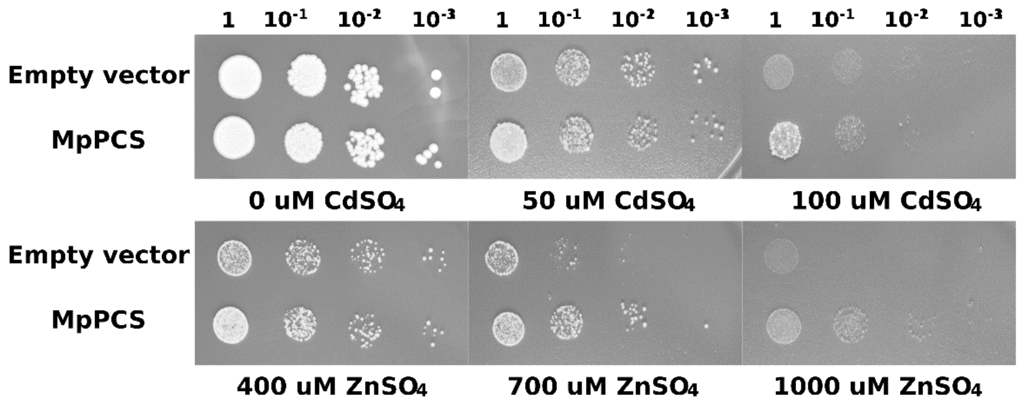
1
10-1
10-2
10-3
Empty vector
MpPCS
0 uM CdSO4
50 uM CdSO4
100 uM CdSO4
400 uM ZnSO4
700 uM ZnSO4
1000 uM ZnSO4

Figure S1

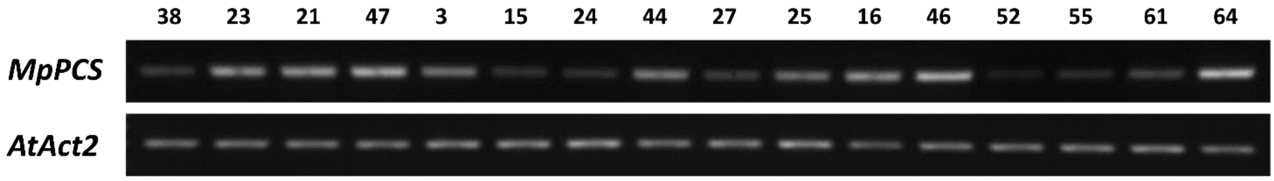

Figure S2

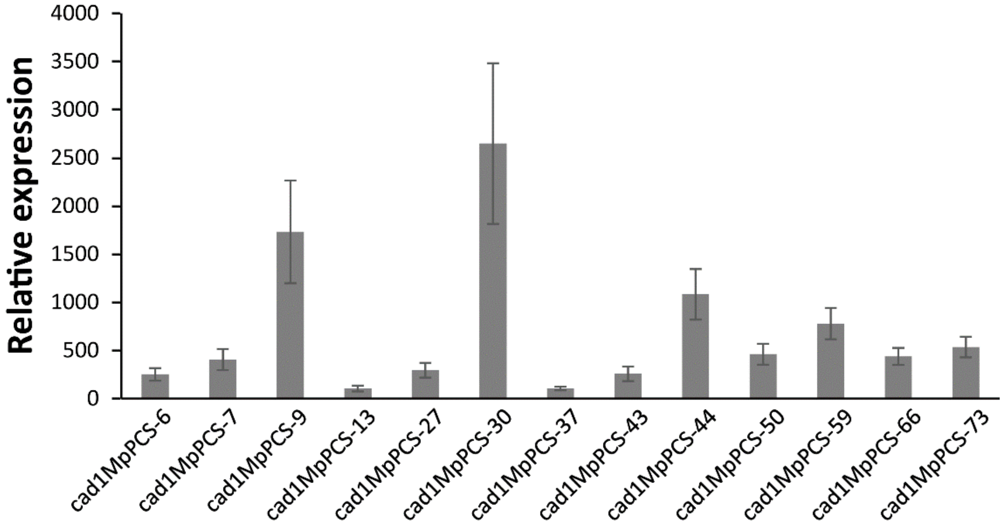
4000
3500
3000
2500
2000
1500
1000
500
0
Relative expression
cad1MpPCS-6
cad1MpPCS-7
cad1MpPCS-9
cad1MpPCS-13
cad1MpPCS-27
cad1MpPCS-30
cad1MpPCS-37
cad1MpPCS-43
cad1MpPCS-44
cad1MpPCS-50
cad1MpPCS-59
cad1MpPCS-66
cad1MpPCS-73

Figure S3

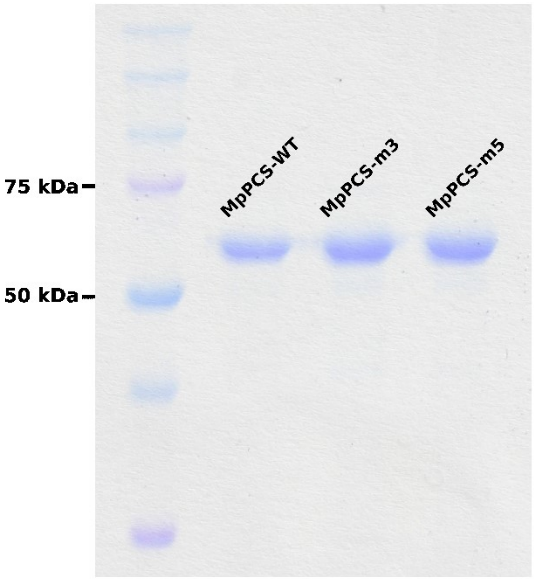

Figure S4

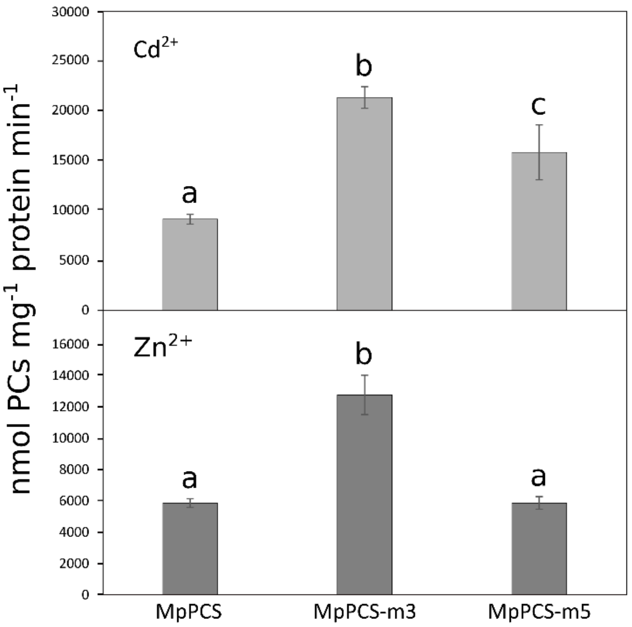

30000
25000
20000
15000
10000
5000
0
Cd2+
a
b
c
16000
14000
12000
10000
8000
6000
4000
2000
0
Zn2+
a
b
a
nmol PCs mg-1 protein min-1
MpPCS
MpPCS-m3
MpPCS-m5

Figure S5
